# Diagnostic Overshadowing and Other Challenges Involved in the Diagnostic Process of Patients with Mental Illness Who Present in Emergency Departments with Physical Symptoms – A Qualitative Study

**DOI:** 10.1371/journal.pone.0111682

**Published:** 2014-11-04

**Authors:** Guy Shefer, Claire Henderson, Louise M. Howard, Joanna Murray, Graham Thornicroft

**Affiliations:** Health Service and Population Research Department, Institute of Psychiatry, Psychology and Neuroscience, King’s College London, London, United Kingdom; Harvard Medical School, United States of America

## Abstract

We conducted a qualitative study in the Emergency Departments (EDs) of four hospitals in order to investigate the perceived scope and causes of ‘diagnostic overshadowing’ – the misattribution of physical symptoms to mental illness – and other challenges involved in the diagnostic process of people with mental illness who present in EDs with physical symptoms. Eighteen doctors and twenty-one nurses working in EDs and psychiatric liaisons teams in four general hospitals in the UK were interviewed. Interviewees were asked about cases in which mental illness interfered with diagnosis of physical problems and about other aspects of the diagnostic process. Interviews were transcribed and analysed thematically. Interviewees reported various scenarios in which mental illness or factors related to it led to misdiagnosis or delayed treatment with various degrees of seriousness. Direct factors which may lead to misattribution in this regard are complex presentations or aspects related to poor communication or challenging behaviour of the patient. Background factors are the crowded nature of the ED environment, time pressures and targets and stigmatising attitudes held by a minority of staff. The existence of psychiatric liaison team covering the ED twenty-four hours a day, seven days a week, can help reduce the risk of misdiagnosis of people with mental illness who present with physical symptoms. However, procedures used by emergency and psychiatric liaison staff require fuller operationalization to reduce disagreement over where responsibilities lie.

## Introduction

People with mental illness suffer higher rates of physical illness and are more likely to die prematurely as a result of physical illness than members of the general population [1]–[4]. For example, people with mental illness, and with schizophrenia in particular, are more likely to have diabetes [5]–[6], coronary heart disease, stroke or respiratory disease [4], HIV infection and hepatitis, osteoporosis, altered pain sensitivity, dental problems, and polydipsia [6], than members of the general public.

People with severe mental disorders have between four to seven- fold higher mortality rates than the general population [7]. One study found that people with schizophrenia or bipolar disorder score twice as high (where high scores mean lower life expectancy) as persons without a record of psychiatric admission or outpatient contact, on the Charlson Comorbidity Index which predicts the ten-year mortality for a patient who may have a range of comorbid conditions [1]. A review of studies about life expectancy of persons suffering from schizophrenia, mainly from high income countries, have found that their lives are between 10 and 25 years shorter than those of the general population. The same review also identified lower life expectancy in people with other types of mental illness such as unipolar depressive disorder, bipolar affective disorder and schizoaffective disorder [8]. While suicide and other unnatural causes (such as accidents) account for some of this gap, at least 60 percent of premature deaths of people with mental illness are a result of natural causes, with the single most common cause being cardiovascular disease [9]. There is also a higher case fatality rate among people with severe mental illness suffering from cancer [10]–[12].

Various explanations have been suggested for the higher rates of comorbidity and premature death from physical illness. Until recently, most of these explanations have focussed on the patient, the illness and social and economic impact of the disease upon other aspects of the patient’s life. More specifically, these explanations include side effects of medication, failure to seek early treatment or to seek it at all, difficulties in managing medication and complying with treatment more generally, unhealthy life-style and poor economic conditions affecting access to healthcare services [2], [6], [9].

More recent studies have pointed to the contribution of systemic disparities in diagnosis and treatment to the disparity in physical health of people with mental illness and the rest of the population [13]–[14]. Such possible contributions can be indicated from service users’ complaints [15] and from evidence of disparities in treatment – for example, in hospitalisation and pathology tests for diabetes [5], [16]–[17], coronary re-vascularisation procedures and in basic health assessments such as blood pressure monitoring [18]–[19], as well as in screening for cancer [10].

One form of systematic disparity to attract growing attention in recent years is a form of discrimination by healthcare professionals known as ‘diagnostic overshadowing’. In this context, ‘diagnostic overshadowing’ refers to the process by which a person with a mental illness receives inadequate or delayed treatment on account of the misattribution of their physical symptoms to their mental illness [14]. A similar problem has been investigated with respect to the attribution of physical illness and particularly symptoms of mental illness to the cognitive impairments of people with learning disabilities [20]–[22]. However, despite some evidence of diagnostic overshadowing provided by users of mental health services [15], very little research focused on the diagnostic overshadowing of people with mental illness until recently. Accordingly some have called for the development of a body of research in this area and pointed to Emergency Departments (EDs) as a setting that required special attention [14].

Following this call, a group of researchers including some of the present authors, undertook a preliminary exploration of diagnostic overshadowing by conducting a qualitative study based on interviews with clinicians in a London general hospital [23]. The aim was to explore the views and experiences of ED doctors and nurses regarding diagnostic overshadowing: what factors made it more or less likely to occur; its frequency; and what might be done to reduce the likelihood and rate of its occurrence. The study identified eight determinants of differential outcomes related to three major problem categories: problems in eliciting a history, problems in the ED setting, and problems related to labelling and stigma.

There were some limitations to these findings, however, given that the data were gathered in one hospital only and that only ED staff were interviewed. We therefore designed a qualitative study on a larger scale which explores diagnostic overshadowing in four hospitals from the perspectives of both ED staff and psychiatric liaison team members. Using the insight of psychiatric nurses and psychiatrists working with EDs, in addition to that of ED staff, the aim of this study is to establish a deeper and more generalisable understanding of the challenges involved in the diagnostic process and the dynamics that affect the risks of misdiagnosis or delayed treatment in this context.

## Materials and Methods

This is a qualitative study based on semi-structured interviews with Emergency Medicine doctors and nurses as well as psychiatrists and psychiatric nurses in four hospitals in south London during 2012 and 2013.

### Ethics

The study was fully approved by the Psychiatry, Nursing and Midwifery Research Ethics Committee of King’s College London. Access of researchers to participating hospitals was approved by the Research and Development departments of each of them. Written consent was obtained from each participant who expressed interest to take part in the study. The consent form was approved by the Psychiatry, Nursing and Midwifery Research Ethics Committee of King’s College London, as part of their full approval of the study.

### Sample and settings

The four hospitals in the study are all located in London. They are among the capital’s biggest hospitals and their EDs are among the busiest in the country, with ED attendance level of up to 350 patients a day in the biggest and 180 in the smallest. In addition, all four hospitals have a high intake of about 3–4 percent of patients with presentations related to mental illness. In each hospital, psychiatric liaison team members, made of psychiatrists and psychiatric nurses, are available for referrals and consultation 24 hours a day, for seven days a week. According to the Guidance for Commissioners of Liaison Mental Health Services to Acute Hospitals [24], liaison teams may have various roles of mental health assessment, providing advice and training for acute staff and assisting acute staff with assessment and management of some group of patients. However, the main (and, for some, the only official) role of the psychiatric liaison units in EDs as understood by most of the interviewees in this study, is to assess the mental health of patients whose presentation is related to mental illness and to decide about further referral and treatment, which may include admission to a psychiatric hospital if necessary. On average 5–12 referrals from ED were made over a twenty-four-hour period in each of the four hospitals. The population attending these hospitals is diverse.

We sought a purposive sample of ten participants from each ED and ten from each of the psychiatric liaison teams. Any nurse or doctor working in the ED for more than a month was considered eligible. Recruitment was administered initially by a member of staff in each department that agreed to act as a local collaborator and approached all staff in the department in general emails about their willingness to take part, providing them with an information sheet about the study. Staff members who were interested contacted the research team and interviews were set. Two participants who initially contacted the research team in order to be interviewed later declined because of heavy workload. None of the participants who were interviewed withdrew their consent during or after the interview. The data collection method used was semi-structured interviews. Interviews took place between October 2012 to October 2013 and each lasted about an hour, one interview only with each participant. Interviews were conducted in private offices in the Institute of Psychiatry psychology and neuroscience or in the hospitals, where only the interviewer and the interviewees were present in order to ensure privacy and confidentiality. There was no official pilot stage, beyond the preliminary study cited above [23], although issues that were raised by staff in the first five interviews were discussed at more length in the following interviews.

### Data collection

All the interviews were audio-taped. All interviews were conducted by GS, a Post-Doctoral Researcher with extensive experience in conducting qualitative interviews. The interviewer did not know any of the interviewees beforehand and did not have any working relationships with any of the hospitals. Participants were asked about the nature of the diagnostic process for people with mental illness presenting in ED. They were asked how the diagnostic protocol for a physical illness differed, if at all, between patients with and without a mental illness. Participants were also asked to describe any emergency case they recalled where an existing psychiatric disorder interfered with the diagnosis of a physical illness. Staff were asked about the relationships between ED and the psychiatric liaison team, about different aspects of working together, and about their impact on the diagnostic process. In addition, all participants were asked for recommendations to optimise the diagnosis of people with mental illness who present with physical problems.

(The interview script can be found in Text S1).

### Data analysis

The interviews were fully transcribed verbatim. Thematic analysis was used to analyse the data. The transcriptions were coded using NVivo software. NVivo is a qualitative data analysis (QDA) computer software package which allows users to classify, sort and arrange information; examine relationships in the data; and combine analysis with linking, shaping, searching and modelling. Analysis involved the following stages: data management which included the following sub stages: (1) familiarization with the data and immersing in the data, including reading transcripts and notes and listening to the audio dialogue in order to extract main themes and ideas; (2) thematic framework development, identifying the key issues and concepts present in the data and creating a coding tree which is the organisation of set of headings in which people’s views experiences and behaviours can be organised in [25]. The coding tree was conducted both inductively, based on the data and deductively based on the research questions; (3) indexing the data – sorting all the parts of the data and are about the same thing and belong together [25]. The thematic framework included the categories ‘Actual cases/consequences’ (with six sub-categories such as ‘death’; ‘irreversible damage to health’; and ‘near misses’) and ‘Views about the scope of diagnostic overshadowing’ – both these categories were later clustered into the head category ‘Scope and occurrences’. Three other categories were ‘Patients and presentations factors’ (with subcategories such as ‘complex presentations’; ‘difficulties in eliciting information’; ‘refusal to be examined’ and ‘other challenging behaviours’), Environmental issues, and Staff attitudes, these all formed the head category ‘Possible causes’. The data management was followed by the interpretation stage - non computerised reviewing and making sense of the data which is sorted and indexed to categories [25]. This included defining the main concepts and recurrent themes in the dialogues, mapping in what ways the different parts of the data are connected in order to try and explain the mechanisms that lead to misdiagnosis or delayed treatment in this context as well as creating typologies and hierarchies of factors and approaches responsible affecting and explaining diagnostic overshadowing [25]. The coding was conducted primarily by GS but verification and refinement of coding throughout the process was done in discussions with CH.

### Extending the focus of the research

While our original aim was to explore the scope and nature of diagnostic overshadowing through the analysis of the clinicians’ accounts, during early interviews and data analysis it became clear we needed to extend our focus to embrace the overall diagnostic process of people with mental illness presenting in ED with physical symptoms. It became evident that diagnostic overshadowing and stigmatising views affected the diagnostic process for some patients, but this was typically only one factor and it is hard to discuss its impact on the diagnostic process without referring to other factors at work. Below we describe other challenges involved in this diagnostic process.

## Results

### Sample

Thirty-nine clinicians were interviewed, 18 doctors (46%) and 21 nurses (54%). Nineteen participants were ED staff (49%) and 20 were psychiatric liaison team members (51%). Eighteen were male (46%, out of which 6 were nurses and 12 doctors) and 21 female (54%, out of which 6 were doctors and 15 were nurses). Twelve of the doctors (67%) had senior appointments (as registrars or consultants) and 6 (33%) were junior trainees. Three of the psychiatric nurses also had general nurse training. Length of professional experience varied with the range being between 6 and 24 years since qualification for the nurses (mean = 12.8) and between 1 and 23 for the doctors (mean = 11.2). Thirteen of the doctors (68%) and all the nurses had more than 5 years of professional experience since qualification. All the nurses and the 12 senior doctors (67% of the doctors) have been working in their department for at least two years. The 6 trainee doctors (33%) have been working in their department for periods of between 3–18 months. There was at least one nurse and one doctor from each ED and from each psychiatric liaison team. Most interviewees were white British, although the sample included participants from other white, Asian and African Caribbean backgrounds.

### The scope and consequences of misdiagnosis

We asked all interviewees to try and recall cases in which a psychiatric disorder interfered with the diagnosis of physical illness. Not all staff recalled such cases: six of the ED staff (15% of the whole sample) and three of the psychiatric liaison team participants (8% of the whole sample) did not recall any, although they were asked about and discussed other aspects of the diagnostic process of people with mental illness who present in EDs. All other participants (77%) reported one or more incidents in which psychiatric disorder led to misdiagnosis, or delayed examination or treatment with a varied degree of seriousness and with a range of consequences. The two most severe cases reported in this context involved the death of patients who refused to be examined and staff failed to conduct any assessment of their mental capacity to refuse treatment. There were two other cases of death that staff suspected might have been in consequence of psychiatric disorder interfering with the diagnosis of physical symptoms. However, their suspicions could not be verified as they did not have enough information about the case or it was not investigated. In these two cases, a patient with mental illness was sent home from the ED without the diagnosis of a risky or urgent condition, and died several days afterwards. There were also five cases in which delayed diagnosis led to irreversible, long-term damage to the patients’ health. The two most serious ones ended with the patient becoming paraplegic in one case and having some of his intestines removed in the second one.

There were reports of more frequent ‘near misses’, a total of eleven specific cases. A typical ‘near miss’ happened when the ED staff ‘medically cleared’ a patient and referred him to the psychiatric liaison staff for mental health assessment, whereupon the latter group insisted upon further physical examinations during which an organic problem was diagnosed. In other eight cases reported by interviewees no lasting damage was caused by the delayed diagnosis but the patient suffered considerable discomfort, such as having to go back and forth between the psychiatric ward and ED, sometimes more than once.

We also asked participants for their overall assessment of the scope of the risk of misdiagnosis or delayed treatment of people with mental illness presenting with physical symptoms. Despite the range of actual cases described above, 17 participants (44%, 5 of them were psychiatric staff members) did not think the risk of misdiagnosis or delayed treatment for physical illness was higher in their hospital for patients with history of mental illness than it was for patients with no mental illness.

Fifteen participants (38%, 9 of whom were staff from the psychiatric liaison teams) thought that there were some specific risks of misdiagnosis or delayed treatment for people with mental illness presenting with physical symptoms in EDs but that those risks were mainly limited to complex and relatively uncommon presentations or to patients who refused examination or treatment.

Seven participants (18%, 6 of whom were staff from psychiatric liaison teams in two out of the four hospitals) were more critical of the diagnostic process for patients with mental illness. One of the most critical staff members in one of the hospitals commented:

*The general problem that we have in the ED here, which we didn’t have with or I have not had with other EDs, is as soon as they [the patients] present with mental health, they [the ED staff] are not interested. A psychiatric nurse*

Other critical interviewees did not go as far as arguing that ED staff were ‘not interested’ but also suggested that some ED staff in their hospital had an ingrained attitude problem and that the risk was therefore not limited to exceptional set of circumstances.

### Factors increasing the risk of misdiagnosis

The determinants described by participants as contributing to the risk of misdiagnosis or delayed treatment of patients with mental illness presenting with physical symptoms can be divided into two groups. One group can be identified as ‘direct causes’ – mainly causes that have to do with the nature of the presentation or the behaviour of the patient. These factors make some presentations both more complex and more risky than others and these were present in most of the actual cases of misdiagnosis which staff reported to us. The other group of factors can be identified as ‘background factors’. These are not factors that stem from the presentation or from the patient’s behaviour but can either affect their behaviour externally (for example, the chaotic nature of the environment) or can affect the ability and motivation of the medical staff to dedicate the time and effort required for a thorough examination (for example, time pressures or staff stigmatising attitudes). The direct causes are discussed here first, followed by the background causes.

### Complex presentations and medical clearance

When patients arrive in ED with a relatively easy-to-diagnose physical complaint such as a broken bone, and their behaviour is not challenging, the risk of misdiagnosis is small. In contrast, more complex presentations considerably increase the risk of misdiagnosis. A typical complex presentation is one that may look like episodes of mental illness because the patient is confused, disorientated or depressed, whereas in fact they are a result of organic cause (or a combination of an organic and psychiatric causes). Another form of presentation that can fall into the category of complex presentations is the one that is known as medically unexplained symptoms (MUS). When initial physical examinations provided no indication regarding what was the medical problem, it was ‘easier’ where patients with mental illness were involved, to assume that the problem was related to mental illness. This is how the following interviewee explained it when asked if there are any differences between the diagnostic process of people with and without history of mental illness:

*There is a difference when the doctors can’t diagnose any reason for the physical complaint. If somebody with some sort of psychiatric disorder comes in and they [ED staff] couldn’t identify what is the problem, they would just tell them, “we’ve done bloods, we’ve done check, we can’t find anything wrong to explain”. There’s a high likelihood they’d then refer us, to us, to try and explain what is the problem. A psychiatric nurse*

The ED staff has responsibility for deciding whether there is any organic cause for the presentation, or whether the patient is ‘medically clear’, and has to be seen by the psychiatric staff for mental health assessment. Most of the psychiatric liaison team interviewees believed they were not supposed to be involved in this process as this is the ED staff expertise.

Procedures related to medical clearance were one of the most common causes of tension and disagreement between ED staff and psychiatric liaison teams. Some psychiatric liaison team participants argued that ED staff were not always aware of possible organic causes for presentations that may appear as an episode of mental illness. ED staff insisted they were aware of the risk that presentations of confused or disoriented patients have organic causes and conducted tests to address such a risk. They opposed, however, conducting intrusive examinations where indications for such a risk were, in their view, very remote, emphasising they cannot reduce risk of misdiagnosis to zero. They also warned against a simplistic assumption that certain examinations such as blood tests would always provide a simple and rapid pointer to the nature of the presentation.

Overall ED staff were more inclined to accept a request for further medical clearance if it named the specific procedure or examination to be conducted and explained why it may contribute to more accurate diagnosis. The chances that such a request would be accepted were higher if it came from a psychiatrist or from a nurse who had had a general nurse training in addition to the psychiatric one. Psychiatric nurses without dual training were less confident about making specific requests for further examinations, apart from the general request for medical clearance which was not receive that well by the ED staff. As one of the ED doctors explained: “Sometimes, when people have said, ‘Have you done bloods?’ I’ve replied ‘Looking for what?’ and we drew a blank”.

In most cases, when ED accepted the need for further examinations, no organic disorders were identified. Such cases might have reinforced ED staff views that psychiatric liaison teams are too quick to make such demands. However, as already noted, participants recalled eleven specific cases where a request for medical clearance ended with a positive result and required further medical examination. Among those were a patient with liver failure, which was diagnosed after the psychiatric nurse insisted on blood tests; and a patient with herpes encephalitis which was diagnosed after a psychiatrist noticed a rash on the patient’s face and insisted on a lumbar puncture. These two cases could have developed into a life-threatening situation if not identified early. Other cases included myxoedema, delirium, overdose of paracetamol (acetaminophen) and renal impairment complications that were all diagnosed thanks to the insistence by psychiatric liaison staff that patients referred to them for mental illness assessment were sent back for further physical examinations.

### Frequent attendees

A particularly risky situation in the view of participants related to complex presentations involved patients with unexplained physical symptoms who attended EDs frequently. Each of the four EDs had a number of frequent attendees who showed up on a regular basis with a similar presentation of medically-unexplained symptoms. The risk was that the symptoms did have an explanation that could be diagnosed and treated. It was also possible that sooner or later they may arrive with a more urgent or even life-threatening problem which would be ignored because of the assumption that it was the same minor unexplained (or, for some staff, ‘imaginary’) symptoms that has caused the patient to attend on a previous occasion. Most interviewees from all hospitals were well aware of the possibility of the ‘boy who cried wolf’ syndrome. While there were some arrangements in place to address that risk, and while this risk could be minimised, it was widely viewed that it was unrealistic to expect staff to be as alert with a frequent attendee as with a patient who arrived at ED with a first presentation.

In some cases, a lengthy and thorough examination, as well as commitment to communicate in spite of the difficulties, enabled ED staff to diagnose a physical problem that had been ignored by other staff and which could reduce the frequency of future visits, as in the following case described by one of the interviewees:

*One of our patients who had attended ED over fifty times this year, always under the influence of alcohol and always requesting strong analgesia, has underlying mental health problems such as depression and anxiety. Eventually I thought I needed to spend a bit more time with him and, actually, when I was able to put aside some time and spend some time with him, it turned out that, actually, he’d been getting a lot of sciatica and neurologic pain in his leg and the alcohol and the request for tramadol was, actually, just for pain relief for his back pain and his sciatica. So, we then addressed that and, actually, since then, his attendance has dramatically reduced. An ED staff member.*

### Difficulties in communication and challenging behaviour

As the above quotation suggests, in some cases complex presentations may become even more complex because of difficulties in communication or because of patients’ behaviour. Some patients who are mentally ill may have difficulties in communication and these can prevent or delay the diagnosis even with relatively straightforward, easy-to-diagnose physical symptoms:

*We had a man who was behaviourally disturbed and he was just screaming out in pain and they all thought he was psychotic, and I said “What’s wrong?” and I looked down at his foot and it was really, really swollen and nobody… Apparently, he had an accident and fractured his foot. Nobody had spotted it so they just referred him straight to me. They’d medically cleared him and I’m a general nurse, so I said “Hang on a minute. Something’s not right with his foot.” And they X-rayed it. It was completely shattered to bits. A psychiatric nurse*

In many cases, however, diagnosis required not just physical examination but a detailed description from the patient about the nature and history of the problem. With patients who suffered from disorientation, disorganised thinking or delusions, it could become difficult to elicit details. An example of difficulty in communication was provided by one of the nurse’s description of a patient who complained about being attacked by his legs. Because he described it in this delusional style it wasn’t initially taken seriously, but after a thorough examination it was found that the person suffered from insufficient blood-flow to the lower limbs, causing pain on walking. As these cases demonstrate, a patient and detailed investigation accompanied by a thorough examination could help in identifying what the physical problem might be.

A more challenging and risky behaviour in this regard was refusal of the patient to permit certain medical procedures (especially those involving needles – for phlebotomy, for example). As noted above, the two cases resulting in death and two cases that ended with irreversible damage to health were caused, according to interviewees, because patients in critical but treatable conditions refused to be examined. In both death cases there were no records showing that staff conducted capacity assessment in order to decide whether they did not have capacity to decide about being examined or treated in order to save their lives.

The following account points to a lack of clarity about whose role it is to conduct the capacity assessment and the heavy price paid for disagreement in one of the cases ending in death:

*This chap had taken quite a large overdose. We got our senior registrar to assess and his attitude was: “He’s a psychiatric patient. I’m not getting involved. Psych need to come and deal with him.” And the psychiatry were like, “Well, he’s not been medically cleared. We can’t get involved and any doctor can do a capacity assessment, so we’re not going to come and do your capacity assessment.” Unfortunately, the guy died on our clinical decisions unit. An ED staff member*

This quotation demonstrates the huge responsibility involved in conducting capacity assessments and also some lack of clarity and confusion about the roles of ED and the psychiatric liaison teams in this context. Some procedures have changed in this and some of the other hospitals following this incident and the interviews revealed consensus among all staff that it is the responsibility of ED to conduct these assessments. Several psychiatric liaison team interviewees remarked that senior doctors in the EDs were more skilled in conducting capacity assessments than junior doctors and those on the wards and that the main problem was the lack of experience of junior doctors in short term posts.

A small minority of patients with mental health problems not only refused treatment but displayed other challenging behaviour. Such behaviour could seriously disrupt communication with the patient but also the work of the whole ED. In the opinion of some of the psychiatry liaison interviewees, this can lead to short-cuts in examinations and observations that might lead to diagnoses that overlook physical problems. On the other hand ED staff felt that the psychiatric liaison staff sometimes failed to understand the challenges involved in such situations and the detrimental impact on the whole ED if they are not dealt with urgently.

### Background factors

While the nature of the presentation or the behaviour of the patient were typically regarded as the direct causes complicating the medical procedures and increasing the risk of misdiagnosis, there were also important background factors that either put extra pressure on staff or patients or hindered staff and reduced their ability to diagnose or provide effective treatment.

### The crowded nature of the ED environment

There was a relative consensus among the participants that the ED is not an ideal environment for some patients with mental health problems. It is noisy, crowded, it is occupied by many people who are nervous or upset, and for people who do not work there it may seem chaotic. Being in such an environment can be particularly challenging for people with mental illness and exacerbate existing symptoms of delusions of reference, anxiety or agitation. As one of the staff members explains:

*It’s a terrible environment for people with mental illness. The waiting room is horrific for them to have to wait in that environment. Once they get into the department, it’s a very busy department and they are having to sit in very busy areas and people coming…it’s not good for somebody with a mental health problem to be in there. A psychiatric nurse*

This aspect of the diagnostic process can make some of the tasks to be carried out by ED staff, whether in communication or treatment, more difficult and it can impose extra pressure on staff to move patients to a quieter environment.

### Time pressure and targets

The triage process in EDs together with relatively strict targets on the time patients should stay there – 4 hours in all the EDs in the UK – put constant pressure on staff to move patients on. These time pressures did not allow, according to some staff, the time required to conduct a thorough examination of a patient with mental illness:

*Time puts a lot of pressure on emergency staff. If they had more time, much of these conflicts can be addressed rather easily. Because there is a long waiting time, by the time a healthcare professional conducts a thorough assessment of a patient, some time would have passed so the clinician would be under pressure to make a quick decision. This means not sufficiently doing full scale of investigations, not ringing family or not exploring aspects in detail, purely because of time restraints. Sometimes, as a result of that, a case may be presented as a straightforward mental health problem but, in fact, when we, the psychiatry team, revisit the case, we identify from the history that there is a physical health concern and that person needed more investigations. A psychiatrist in the psychiatric liaison team.*

### Stigmatising attitudes

Most participants described the attitudes of emergency staff as non-stigmatising and suggested that stigmatising views were held be a very small minority of staff in some of the hospitals. Participants thought that most ED staff had very good communication skills, better than staff in the wards, and were able to communicate well with different groups of patients. Experienced staff also argued that attitudes had improved considerably over recent years and were better than in other hospitals they had worked in. Nevertheless, references to what can be perceived as stigmatising attitudes, some of them highly stigmatising, were made by some of the interviewees.

In some cases, disrespectful comments were made directly to patients. Such comments, in addition to their offensive nature, could lead to short-cuts in diagnosis:

*There was one patient about whom the nurse said, “I don’t believe anything she’s saying. She’s one of the weirdest women I've ever met. She said that she’d come in with all of these problems and I don’t believe her.” And she didn’t do a lot of the observations.” She hadn’t taken an ECG and she didn’t want to take bloods without me being there. And so I went to speak to her, the patient, and she said that she’d had some horrible problems over at [another] hospital. I ended up calling [the other] hospital who, actually, corroborated all of her stories and, actually, it was worse than what she’d made it seem and she had to go to [one of the units in the hospital] for a long time. A psychiatric nurse*

In some cases, the problem was not in expressing stigmatising views as such but in the way that the mental illness dominated the discussion between medical staff, even when the patient arrived with a presentation of physical symptoms:

*One patient told me […] that every time he came, someone would say, “He is a known schizophrenic”. That is how they would start presenting his case to each other. He didn’t like that […] And he feared, and his fear was true, apparently, according to his experience [with regard to reoccurring abdominal pain], that by saying that there is a mental health problem, people overlook the physical health. A psychiatrist*

Other interviewees commented that some staff kept the interaction time with patients with mental illness, especially those more difficult or less pleasant to communicate with, to the minimum possible. This meant, for example, that some of these patients were subject to only one set of basic observations, while a person with no mental illness who spent the same time in the ED would undergo a second or third set of observations throughout their stay in the ED.

What made this minimal interaction with some ‘difficult’ patients more acceptable was the notion of some ED staff, as reported by some of their colleagues, that people with mental illness histories ‘belonged’ to the psychiatric liaison team and were ‘their patients’, hence, their stay in the ED is only a temporary station before they move to the psychiatric liaison team where they belong. Several psychiatric nurses reported how ED staff, when talking to psychiatric staff about patients waiting in the ED, referred to them as ‘your patient’. A psychiatric nurse recalled how an ED staff member commented, ‘I just resent the fact that I’m dealing with your patients when I should be looking after my own’.

Some ED staff suggested the pressure to move patients to the department where they ‘belong’ is not unique to patients with psychiatric history but part of the role of the ED as a triage unit. However, this pressure to move on patients to where they ‘belonged’ seemed to be particularly strong when it came to patients with a history of mental illness.

*The psychiatric team can often be seen as a dumping-ground for people who have a mental health problem. So, as soon as it looks like it's a mental health problem then people, kind of, switch off and say “We have to do what we can so that they will take them away so that we can deal with other people.” And I think there is that mentality that goes on a bit.*

*Mental health patients, they go to a special cubicle. So, already, there is a ‘section’: mental health. It's not physical. So the staff are creating an idea about what the problem is without a full assessment. The barcode, as I call it, on the forehead is already booked. I can understand why it happens but I think, still, it's wrong. An ED doctor.*

The risk with this approach, as the above quotation demonstrates, is that as a result of the presumption that these are psychiatric patients, the diagnostic process will be rushed and the focus will move to people who more ‘clearly’ belong in the ED.

Again, many interviewees commented that such a ‘them and us’ division was much worse in the past, in other wards, or in other hospitals they have worked in before, and that overall relationships between the two departments in each of the four hospitals were good. However, a basic sense that these patients are part of a different group that belong elsewhere did not completely disappear.

## Discussion

This study confirmed that diagnostic overshadowing can lead to misdiagnosis of people with mental illness who present in ED with physical symptoms. Interviewees reported a series of specific cases, with varied degree of seriousness, in which mental illness interfered with a diagnosis of physical problem. We also found evidence of a hierarchy of risk between the factors which may affect the diagnostic process in this context. We distinguished between the more direct causes of complex presentations and difficulties in communicating or challenging behaviour by patients, and the background factors such as time pressures and staff stigmas which may lead to wrong or delayed diagnosis and treatment in this context.

The range of cases reported by participants in this study in which a psychiatric disorder interfered with the diagnosis of a physical problem, included two cases of death and five cases of irreversible long-term damage to people’s health, suggests that special challenges are involved in this process, and that failure to address them can lead to considerable number of misdiagnoses and delays in treatment, with severe and detrimental consequences to health.

In a small number of cases, the stigmatising views of staff had been a serious factor in preventing the timely and accurate diagnosis of a physical problem. However, on the basis of views of most interviewees it seems that, more typically, a stigmatising attitude is only one of several contextual factors and that it exists in some but not all cases of the misdiagnosis of physical symptoms among patients with mental illness. The main risks for misdiagnosis are related either to the complicated nature of the presentation or to difficulties in communication or challenging behaviour by some patients. This finding is consistent with the prediction of researchers that ‘diagnostic overshadowing’ is unlikely to be simply the result of bias or of discriminatory attitudes and that a range of other factors related to the communication between doctor and patient may also be relevant [14].

It is important to emphasise that by drawing attention to the contribution of complex presentations or challenging behaviour we do not shift the responsibility for misdiagnosis in such cases from the medical staff to the patients. It is still the full responsibility of ED staff to make correct diagnoses, even if the presentation is complex and also, in the case of people with mental illness, if the behaviour is challenging, as this is part of the medical disorder. Neither of these findings should undermine the importance of background factors and, in particular, of stigmatising opinions and time/target pressures.

While most participants reported that stigmatising opinions were held only by a minority of staff in some hospitals, their existence is still worrying, especially since some of the more critical participants argued that it was enough that one or two dominant staff members held stigmatising views for the whole ED culture to become less mindful of the needs of people with mental illness. It should also be reiterated that stigmatising opinions towards people with mental illness in EDs can be upsetting and demoralising, sometimes even traumatic regardless of their impact on the misdiagnosis of physical illness.

Psychiatric liaison team members have a key role in reducing the risk of misdiagnosis in these circumstances. They may be a last safety net, turning ‘real misses’ into ‘near misses’ by insisting on the further examination of patients referred to them after being medically cleared. The existence of the liaison team is a mitigating factor which does not exist in relation to other vulnerable populations, such as patients with learning disabilities. However, many of the psychiatric nurses lack a general nursing training and therefore their ability to contest misdiagnoses made by ED staff is limited. They do not have a formal role in the ED diagnostic process beyond conducting a mental health assessment. Further, not all hospitals have round the clock coverage by a psychiatric liaison team, as do the hospitals in the present study. Last, while their presence is useful in many ways, it can have adverse consequences, in particular in it creating a reduced sense of responsibility of emergency staff for patients they see as belonging to another team.

### Limitations, strengths and further research

The study has been conducted in four hospitals with a high intake of people with mental illness attending the EDs and with round the clock coverage by psychiatric liaison teams. Although the sample is bigger than in previous studies [23], as with any qualitative study, caution is required before generalisation of conclusion, especially given that the experiences discussed here are based on staff recollection of the past and may be subject to memory bias and other cognitive distortions. Future studies may wish to add observational element in order to capture cases of diagnostic overshadowing when they take place. Also, some of the hospitals operate in close proximity to large psychiatric hospitals, which helps to create a higher level of awareness with respect to mental illness and the stigmatisation of people with mental illness. Additional research in hospitals with no such coverage might reveal even greater cause for concern.

The reported cases of misdiagnosis can shed light on the dynamics and specific risks involved in the diagnostic process. In order to provide an accurate estimation of the frequency of such cases, a more quantitative study should be conducted, covering all cases in certain EDs within defined time-periods. The results of such a study could then be compared with findings of misdiagnosis in patients without mental illness presenting in ED, or of EDs in which psychiatric liaison teams operate only during normal working hours. While there are some limitations in the level of stigmatisation being self reported by ED staff, this limitation was mitigated in this study by interviewing also members of psychiatric liaison teams. Further research may focus also on experiences of patients with history of mental illness in this regard.

### Clinical implications

From our exploration of complex cases it is clear that the procedures used by emergency and psychiatric liaison staff require fuller operationalization to reduce disagreement over where responsibilities lie, for example to reduce unnecessary delays in capacity assessments and in psychiatric admissions, and repeated transfers between the ED and psychiatric units. It also appears that there are simpler cases in which physical conditions such as fractures and other causes of pain are missed due to an assumption that people using psychiatric services ‘belong’ with the psychiatric services. These cases suggest training needs in addition to the need for agreed procedures for more complex cases. While the provision of psychiatric liaison teams may improve the cost-effectiveness of general hospital’s care of people with mental illness [26], an overall conclusion that can be drawn from the current study is that the full potential for improvement in quality of ED care represented by these teams is currently not being realised, for example due to unintended consequences in the form of problems between the psychiatric and emergency teams. In addition to a maximum availability and accessibility of psychiatric liaison team throughout the day and week, the study has some implication for training of ED staff, of procedures regarding parallel assessment by both teams, for the qualification and basic training of psychiatric nurses, for enhancing and structuring communication channels between the two departments and for using specially adjusted written pathway and of access of ED staff to electronic records of mental illness history of patients. In the future we aim to provide more specific list of recommendations and guidelines shaped by professionals interviewed for this study and members of Non Governmental Organisations advocating for better physical health care for people with mental illness and learning disabilities, about how to improve the diagnostic process of people with mental health problems presenting with physical complaints at the ED.

## Supporting Information

Text S1 **Interview scripts used in study.**
(DOCX)
