# Supplementary material for: Diagnostic Overshadowing and Other Challenges Involved in the Diagnostic Process of Patients with Mental Illness Who Present in Emergency Departments with Physical Symptoms – A Qualitative Study
Source: PLoS One. 2014 Nov 4;9(11):e111682. doi: 10.1371/journal.pone.0111682 (PMC4219761; doi:10.1371/journal.pone.0111682)
Supplement: Text S1 — Interview scripts used in study. (DOCX) [file pone.0111682.s001.docx]

**Interview guide S1**

1. **Interview guide for ED clinicians (non psychiatrists):**

The following questions will provide the framework for the one-to-one interview. While questions that are not listed here may be asked in order to follow up on participant responses, the interview will centre on these main questions. The introduction and debriefing statements will be read to participants.

***Introduction***

*The purpose of this study is to find out your views and experiences about establishing the diagnosis among patients who have a severe psychiatric illness who present at the ED with a physical symptom. We would like you to share your views and any experience that you may have had while working in the ED. Everything that you say here will be kept confidential, and your name, and any other identifying information will not be used in any report coming from this research.*

*Can you tell me what your role in the Emergency Department is and what is your daily routine when working here?*

*What would alert you that a patient arriving to the emergency department may have mental health problem.*

*What kind of decisions do you make when someone comes through the door and portray a behaviour that makes you think they have mental health problems?*

*What types of individuals that comes to the emergency department cause you concern? Why?*

*Have you witnessed or been involved in a case where an existing psychiatric disorder interfered with establishing a diagnosis of a physical health problem?*

**If yes**, why do you think this occurred?

Also ask them to describe what happened and what contributed to the misdiagnosis. Probes:

- *What was your initial response?*
- *Did the situation involve other people?*
- *What role did the health care professionals play?*
- *Did you involve other people?*
- *Who did you talk to?*
- *Were there things about the patient that made it hard to establish the diagnosis? Probe for:*
  - *communication problems,*
  - *psychiatric symptoms,*
  - *psychotropic medication side effects*
- *Was there a problem with the role of the professional? Probe for:*
  - *knowledge of severe mental illness or*
  - *attitude towards people with such illnesses*
- *Was there a problem due to the ED setting? Probe for:*
  - *pressure of time,*
  - *lack of privacy or noise*
- *Was there a problem due to patient characteristics? Probe for:*
  - *age*
  - *ethnicity*
  - *gender*
  - *language*
  - *other cultural differences*

Do you think the process of diagnosis differs for patients with a physical presenting complaint and co-morbid psychiatric illness from those with physical presenting complaints only. In what way? Probes:

- *Are there things about people with severe mental illnesses that make it harder to* *establish the diagnosis? Probe for:*
  - *communication problems,*
  - *psychiatric symptoms,*
  - *psychotropic medication side effects.*
- *Does any thing about ED health professionals make it harder to establish the diagnosis? (Probe for:*
  - *level of psychiatric knowledge,*
  - *attitudes,*
  - *comfort around people with psychiatric illness*
  - *professional backgrounds*
- *Do age, ethnicity, gender or language make it harder to establish a diagnosis?*
  - *If so, why?*
- *Have you consulted liaison psychiatry staff?*
- *If so, why?*
- *If not, why not?*
- *Does the ED setting affect people with psychiatric illness differentially? Probe for: pressure of time, lack of privacy or noise?*

***Key questions***

*Overall, do you think that it is more difficult treating a person presenting with a physical complaint who also has a severe mental illness? If so, how?*

*How often do you think psychiatric patients (be more specific as before) experience misdiagnosis due to misattribution of their symptoms, or delay in establishing the diagnosis?*

*What impact does this phenomenon have on patients?*

*What impact does it have on the professionals concerned?*

*What impact does it have on the ED?*

*What impact does it have on other parts of the health service?*

***Transition with example***

*I am now going to give you a sheet of paper which has a real-life example of what took place at the Maudsley Hospital several years ago. I would like you to read the example and ask me any questions you may have about it.*

*Note – The sheet of paper will have the following example on it:*

*A middle-aged woman was referred by her psychiatrist to the Maudsley Hospital Emergency Clinic for treatment of a relapse of a psychotic illness, having presented with disorganised behaviour and self neglect. The patient had a history of breast carcinoma which had been treated several years ago. The patient complained to the Senior House Officer (SHO) assessing her at the Emergency Clinic that she was unable to maintain her balance as she was unsteady on her feet. She was admitted for treatment of a relapse of her psychotic illness. Her unsteadiness got worse throughout the night to the point where she could no longer stand and had fallen down. Dissatisfied with the SHO’s explanation that her fall was behaviour related to the mental illness the ward nurses called the Specialist Registrar on call. When she arrived, the patient was lying down and unable to move her legs. The SpR did a physical assessment and found her to be in urinary retention. Having decided that the most likely cause was developing spinal shock due to spinal secondary carcinoma she contacted the on call neurologist at King’s College Hospital. After an urgent neurology consultation the patient was immediately transferred to the Neurological Intensive Care Unit.*

- *Have you come across any similar situations in the ED? If so, can you describe what happened?*

Probe for barriers and facilitators to determining the diagnosis:

- patient factors
- professional factors e.g. training on psychiatric symptoms, psychotropic medication side effects, communication skills, anti-stigma training)
- ED setting factors e.g. improve privacy, reduce noise

***Ending question***

*What are the most important things that that interfere with diagnosis for physical complaints in people who also have a severe mental illness?*

*What could be done to reduce the risk of this problem occurring in the ED?* Probe for:

- patient factors e.g. advise to bring a carer,
- professional factors e.g. training on psychiatric symptoms, psychotropic medication side effects, communication skills, anti-stigma training)
- ED setting factors e.g. improve privacy, reduce noise

We are trying to come up with a list of recommendations to reduce the risk of diagnostic overshadowing. Based on what you said today what would be your recommendations?

***Follow-up questions*** *will be asked, when appropriate, to gather further information on perceived barriers to diagnosis and suggested interventions to reduce the occurrence of misattribution.*

***Debriefing***

*I would like to thank you for your participation. I also want to restate that what you have shared with me is confidential. No part of our discussion that includes names or other identifying information will be used in any papers, reports, displays, or other publicly accessible documents coming from this research. I want now to provide you with a chance to ask any questions that you might have about this research. Do you have any questions for me? Finally I want to give you my email so in case you have anything else to add and specifically any more recommendations to add – after we end our interview- you can send them to me.*

**B: Interview guide for psychiatric staff:**

The following questions will provide the framework for the one-to-one interview. While questions that are not listed here may be asked in order to follow up on participant responses, the interview will centre on these main questions. The introduction and debriefing statements will be read to participants.

***Introduction***

*The purpose of this study is to find out your views and experiences about establishing the diagnosis among patients who have a severe psychiatric illness who present at the ED with a physical complaint. We would like you to share your views and any experience that you may have had while working in the ED. Everything that you say here will be kept confidential, and your name, and any other identifying information will not be used in any report coming from this research.*

**General**

Can you tell me what is your role as a psychiatric liaison person?

Probe:

- *Are you involved/being notified about every case of a person with existing psychiatric disorder arriving to the Emergency Department?*

What are the main issues that persons with existing psychiatric disorder who appear in Emergency Departments (hospital more generally?) raise?

What can you tell me about the relationships between the psychiatric liaison staff and the non-psychiatric staff?

Probe:

- Are ED staff aware of your role?
- Do ED staff make the best use of consulting you and the other psychiatric staff in order to benefit from your knowledge/experience?
- Are there conflicts/disagreements between the psychiatric staff and other clinicians?

Diagnostic overshadowing

*Have you witnessed or been involved in a case where an existing psychiatric disorder interfered with establishing a diagnosis of a physical health problem? (if more than one, start with the first one, ask all the questions and then move to the next case)*

**If yes**, why do you think this occurred?

Also ask them to describe what happened and what contributed to the misdiagnosis. Probes:

- *What was the initial response?*
- *Who was involved in this situation?*
- *Were you or anyone else from the psychiatric liaison person involved in it? If yes, from what stage and how did you become involved? What did you/they do? If not – why not?*
- *Were there things about the patient that made it hard to establish the diagnosis? Probe for:*
  - *communication problems,*
  - *psychiatric symptoms,*
  - *psychotropic medication side effects*
- *Was there a problem with the role of the professional? Probe for:*
  - *knowledge of severe mental illness or*
  - *attitude towards people with such illnesses*
- *Was there a problem due to the ED setting? Probe for:*
  - *pressure of time,*
  - *lack of privacy or noise*
- *Was there a problem due to patient characteristics? Probe for:*
  - *age*
  - *ethnicity*
  - *gender*
  - *language*
  - *other cultural differences*

Do you think the process of diagnosis differs for patients with a physical presenting complaint and co-morbid psychiatric illness from those with physical presenting complaints only. In what way? Probes:

- *Are there things about people with severe mental illnesses that make it harder to* *establish the diagnosis? Probe for:*
  - *communication problems,*
  - *psychiatric symptoms,*
  - *psychotropic medication side effects.*
- *Does anything about ED health professionals make it harder to establish the diagnosis? (Probe for:*
  - *level of psychiatric knowledge,*
  - *attitudes,*
  - *comfort around people with psychiatric illness*
  - *professional backgrounds*
- *Do age, ethnicity, gender or language make it harder to establish a diagnosis?*
  - *If so, why?*
- *Does the ED setting affect people with psychiatric illness differentially? Probe for: pressure of time, lack of privacy or noise?*

***Key questions***

*Overall, do you think that it is more difficult treating a person presenting with a physical complaint who also has a severe mental illness? If so, how?*

*How often do you think psychiatric patients (be more specific as before) experience misdiagnosis due to misattribution of their symptoms, or delay in establishing the diagnosis?*

*What impact does this phenomenon have on patients?*

*What impact does it have on the professionals concerned?*

*What impact does it have on the ED?*

*What impact does it have on other parts of the health service?*

***Transition with example***

*I am now going to give you a sheet of paper which has a real-life example of what took place at the Maudsley Hospital several years ago. I would like you to read the example and ask me any questions you may have about it.*

*Note – The sheet of paper will have the following example on it:*

*A middle-aged woman was referred by her psychiatrist to the Maudsley Hospital Emergency Clinic for treatment of a relapse of a psychotic illness, having presented with disorganised behaviour and self neglect. The patient had a history of breast carcinoma which had been treated several years ago. The patient complained to the Senior House Officer (SHO) assessing her at the Emergency Clinic that she was unable to maintain her balance as she was unsteady on her feet. She was admitted for treatment of a relapse of her psychotic illness. Her unsteadiness got worse throughout the night to the point where she could no longer stand and had fallen down. Dissatisfied with the SHO’s explanation that her fall was behaviour related to the mental illness the ward nurses called the Specialist Registrar on call. When she arrived, the patient was lying down and unable to move her legs. The SpR did a physical assessment and found her to be in urinary retention. Having decided that the most likely cause was developing spinal shock due to spinal secondary carcinoma she contacted the on call neurologist at King’s College Hospital. After an urgent neurology consultation the patient was immediately transferred to the Neurological Intensive Care Unit.*

- *Have you come across any similar situations in the ED? If so, can you describe what happened?*

Probe for barriers and facilitators to determining the diagnosis:

- patient factors
- professional factors e.g. training on psychiatric symptoms, psychotropic medication side effects, communication skills, anti-stigma training)
- ED setting factors e.g. improve privacy, reduce noise

***Ending questions***

*What are the most important things that that interfere with diagnosis for physical complaints in people who also have a severe mental illness?*

*What could be done to reduce the risk of this problem occurring in the ED?* Probe for:

- patient factors e.g. advise to bring a carer,
- professional factors e.g. training on psychiatric symptoms, psychotropic medication side effects, communication skills, anti-stigma training)
- ED setting factors e.g. improve privacy, reduce noise

We are trying to come up with a list of recommendations to reduce the risk of diagnostic overshadowing. Based on what you said today what will be you recommendations?

***Follow-up questions*** *will be asked, when appropriate, to gather further information on perceived barriers to diagnosis and suggested interventions to reduce the occurrence of misattribution.*

***Debriefing***

*I would like to thank you for your participation. I also want to restate that what you have shared with me is confidential. No part of our discussion that includes names or other identifying information will be used in any papers, reports, displays, or other publicly accessible documents coming from this research. I now want to provide you with a chance to ask any questions that you might have about this research. Do you have any questions for me? Finally I want to give you my email so in case you have anything else to add and specifically any more recommendations to add – after we end our interview- you can send them to me.*
